# Threats to Validity in Retrospective Studies

**Published:** 2012-05-01

**Authors:** Cindy Tofthagen

**Affiliations:** From University of South Florida, College of Nursing, Tampa, Florida

The goal of most interventional studies is to establish a cause-and-effect relationship between the intervention and one or more outcomes. A retrospective study design utilizing existing clinical data is a relatively quick and inexpensive way to collect pilot data, which can be helpful in identifying feasibility issues and designing a future prospective study (Hess, 2004).

The study by Donald, Tobin, and Stringer (2011) discussed on page 178 by Constance Visovsky, PhD, RN, APRN-BC, describes a possible cause-and-effect relationship between acupuncture and chemotherapy-induced peripheral neuropathy (CIPN) using a retrospective design. Studies that attempt to evaluate efficacy of an intervention using a retrospective design are subject to numerous threats to validity, which limit the interpretation and generalizability of the results (Trochim, 2005). These threats are summarized in Table 1.

**Table 1:**
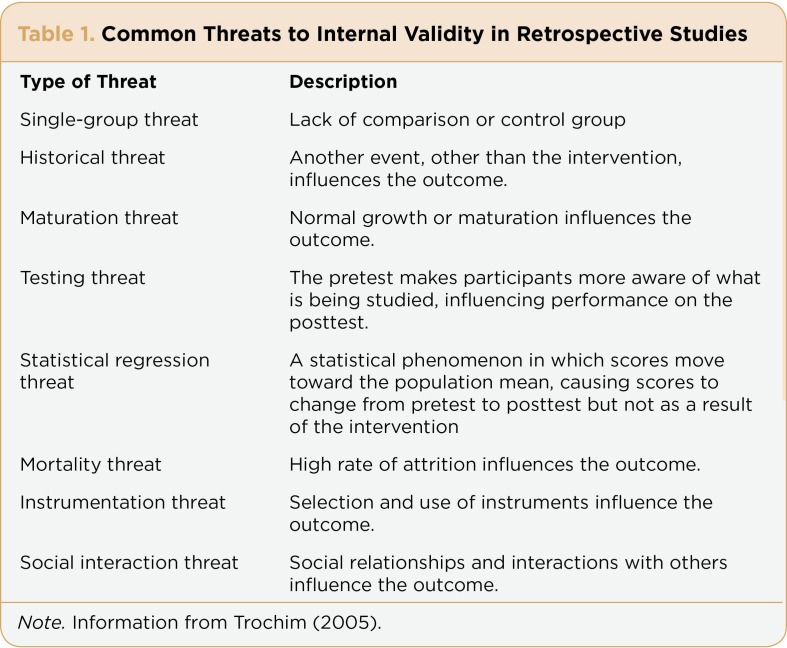
Table 1. Common Threats to Internal Validity in Retrospective Studies

The single-group design often employed in retrospective studies limits the researchers’ ability to determine cause and effect. Although it is usually not possible to include a control group in a retrospective study, whenever possible a control group should be included to help establish the cause-and-effect relationship. Random assignment helps ensure that groups are similar to each other before treatment, so study outcomes cannot be attributed to anything other than the intervention.

## The Donald, Tobin, and Stringer Study

In the acupuncture study, historical threats may have influenced the results. It is unclear how many of the participants had already completed chemotherapy, in which case symptoms may have improved as a result of time and withdrawal of the neurotoxic agent(s). The single-group design in this study limits the researchers’ ability to determine differences between the group who received acupuncture and a similar group who did not receive acupuncture.

## Social Pressure

Patients provided with interventions in clinical settings may feel social pressure to respond positively to the question of whether the intervention provided the desired effect (King & Bruner, 2000). Especially when data are collected by the health-care team providing services to the patient, social desirability bias is a highly plausible explanation for success of the intervention. Similarly, a phenomenon known as the Hawthorne effect may cause participants to report more favorable outcomes because they are receiving closer attention or being more closely evaluated as a result of participation in a study (McCarney et al., 2007). Addition of a control group that would provide participants with a similar amount of attention and evaluation as the intervention group helps control for both social desirability bias and the Hawthorne effect.

## Instrumentation

Another major threat to validity is instrumentation. Although no gold standard exists for measurement of CIPN (Dunlap & Paice, 2006), there are several instruments available for measurement of CIPN that have demonstrated reliability and validity (Almadrones, McGuire, Walczak, Florio, & Tian, 2004; Huang, Brady, Cella, & Fleming, 2007; Tofthagen, McMillan, & Kip, 2011). Eliciting patient preferences and perceptions regarding efficacy is an important aspect of patient care; however, opinions without the use of reliable and valid measures of the outcome variable should be avoided in clinical research. Valid instruments have undergone evaluation to determine the extent to which they actually measure the concept they intend to measure (Waltz, Strickland, & Lenz, 2005). If a blood pressure cuff is used to measure temperature, even though the blood pressure cuff is working as it should, it is an invalid measure of temperature. Reliable instruments consistently produce the same results in the same circumstances. If the blood pressure cuff reveals hypertension on the first measurement and hypotension in the same hemodynamically stable patient 30 seconds later, that blood pressure cuff is an unreliable measure of blood pressure, at least until it can be calibrated and/or repaired. Just as using a valid and reliable instrument to measure blood pressure is important in practice, using valid and reliable measurement tools is equally important in research.

External validity refers to the generalizability of the findings to other samples. In interventional studies for CIPN, generalizing the results to a specific population may not be possible when participants differ in respect to factors that can be expected to influence outcomes such as cancer type, chemotherapy doses, and chemotherapy drugs. It is likely that patients who develop CIPN from one neurotoxic chemotherapy may differ in response from those who receive other types of neurotoxic chemotherapy.

## Conclusions

Retrospective designs provide a vehicle for research using existing data but can be riddled with threats to both internal and external validity. Although a cause-and-effect relationship cannot be determined using retrospective studies, they are useful for providing preliminary data and in guiding the development of future prospective studies.
